# Impact of Cardiovascular Organ Damage on Cortical Renal Perfusion in Patients with Chronic Renal Failure

**DOI:** 10.1155/2013/137868

**Published:** 2013-06-18

**Authors:** Arkadiusz Lubas, Robert Ryczek, Grzegorz Kade, Jerzy Smoszna, Stanisław Niemczyk

**Affiliations:** ^1^Department of Internal Diseases, Nephrology, and Dialysis, Military Institute of Medicine, Ulica Szaserów 128, 04-141 Warsaw 44, Poland; ^2^Department of Cardiology, Military Institute of Medicine, Ulica Szaserów 128, 04-141 Warsaw 44, Poland

## Abstract

*Introduction*. Properly preserved renal perfusion is the basic determinant of oxygenation, vitality, nutrition, and organ function and its structure. Perfusion disorders are functional changes and are ahead of the appearance of biochemical markers of organ damage. The aim of this study was to evaluate a relationship between the renal cortex perfusion and markers of cardiovascular organ damage in patients with stable chronic renal failure (CKD). *Methods*. Seventeen patients (2 F; 15 M; age 47 ± 16) with stable CKD at 2–4 stages and hypertension or signs of heart failure were enrolled in this study. Blood tests with an estimation of renal and cardiac functions, echocardiographic parameters, intima-media thickness (IMT), renal resistance index (RRI), and total (TPI), proximal (PPI), and distal (DPI) renal cortical perfusion intensity measurements were collected. *Results*. DPI was significantly lower than PPI. TPI significantly correlated with age, Cys, CKD-EPI (cystatin), and IMT, whereas DPI significantly depended on Cystain, CKD-EPI (cystatin; cystatin-creatinine), IMT, NT-proBNP, and troponin I. In multiple stepwise regression analysis model only CKD-EPI (cystatin) independently influenced DPI. *Conclusions*. Cardiovascular and kidney damage significantly influences renal cortical perfusion. Ultrasound measurement of renal perfusion could be a sensitive method for early investigation of cardiovascular and renal injuries.

## 1. Introduction

Properly preserved renal perfusion is the main factor conditioning correct oxygenation, thus preserving renal function and structure. It is suggested that disorders in oxygenation of the kidneys are common way for acute and chronic pathological processes [1]. Through the renal cortex flows about 20% of cardiac output, and, thanks to efficient autoregulation, renal oxygenation is partially independent of blood pressure [1, 2]. This mechanism is damaged in chronic kidney disease (CKD), and even an average increase in blood pressure is transferred to renal glomerulus [3]. Disturbances in the form of significant increase, as well as decrease of renal perfusion, cause a dramatic decrease in oxygenation of renal parenchyma [2, 4]. Persistent hypoxia results in tissue damage, interstitial fibrosis, and further reduction of perfusion. This is supported by research by Syversveen et al. in which perfusion of renal cortex in transplanted organs was negatively correlated with the degree of parenchymal fibrosis in the biopsy [5]. Disturbances of perfusion are thus the reason or result of pathologic processes in renal parenchyma. With the progress of CKD, there is a gradual reduction of cortical perfusion from distal to proximal regions, which has been shown in a study by Scholbach et al. [6]. Moreover, in cases of renal diseases of ischemic etiology, reduced tissue perfusion precedes the biochemical markers of the organ function limit [7]. Thus, the detection of perfusion disorders is a more sensitive method of kidney damage evaluation than available standard biochemical tests. Among the performed tests, only the Doppler ultrasound imaging is a cheap, highly accessible, and noninvasive quantitative evaluation of organ perfusion [8]. To this day, in some publications, quantity of renal perfusion has been evaluated based on renal resistive index (RRI) obtained in the Doppler sonography [9, 10]. However, most studies suggest that RRI is not related to perfusion, or even vascular resistance, but it is rather a marker of hypertension control and vascular organ damage [11–15]. RRI value is not specific for either function or renal perfusion [7]. Unlike RRI, ultrasound dynamic assessment of perfusion is a sensitive parameter of changes in renal function, and it may be an important test conditioning early treatment decisions. In the comparative study on a phantom model, the consistency of dynamic ultrasound measurement of tissue perfusion (DTP) by the method proposed by Scholbach was 99,7% [16]. At present, there are no studies on the impact of cardiovascular organ damage (CVOD) signs on renal DTP.

The aim of this study was to evaluate a relationship between the quantity of renal cortex perfusion and markers of cardiovascular organ damage in patients with stable chronic renal failure. 

## 2. Methods

### 2.1. Patients

Seventeen patients (2 F; 15 M; age 47 ± 16; BMI 27.4 kg/m^2^; BSA 1.99 m^2^) with stable chronic renal disease at 2–4 stages and hypertension or signs of heart failure were enrolled in this study (Table 1). Patients were recruited from consecutive subjects who had been admitted to the nephrology department over a one-year period, and they gave informed consents. Exclusion criteria included the following: acute renal and cardiac diseases, hyperkinetic state (pregnancy, fever, etc.), heart failure in NYHA IV stage, atrial fibrillation, connective tissue diseases, vasculitis, diabetes mellitus, amyloidosis, visceral obesity, hydronephrosis, immunosuppressive therapy, systemic cancer, lack of good quality images of renal or heart structures, and inability of reliable heart and renal hemodynamic measurements.

### 2.2. Blood Tests

Blood tests for hemoglobin level and biochemical data including serum creatinine (Crea), cystatin C (Cys) with an estimation of glomerular filtration rate by the CKD-EPI formula (based on Crea and/or Cys), uric acid (UA), NT-pro-brain natriuretic peptide (NT-proBNP), troponin I, and high-sensitive C-reactive protein (CRP) were collected.

### 2.3. Monitoring of Blood Pressure

The 24-hour ambulatory blood pressure monitoring (ABPM-04, Meditech, Hungary) was executed in all patients. Means of 24-hour systolic, diastolic blood pressure (SBP, DBP), pulse pressure (PP), and mean arterial pressure (MAP) were recorded. 

### 2.4. 2D and Color Doppler Sonography

Ultrasound examinations were performed with the Logiq P6 (GE) ultrasound equipment.

To achieve measurements of renal resistance index and renal cortical perfusion, a convex transducer 4 L (2–5 MHz) was used. Longitudinal sections of the right kidneys were examined and recorded. The right kidney was chosen because of better technical access for ultrasound investigation. RRI in segmental arteries was achieved as described before [15]. Mean of 2-3 measurements of RRI performed in the upper, middle, and lower regions of the renal sinus was calculated and considered for statistics. 

The renal perfusion (RP) as an intensity of Color Doppler signal from at least 3 full heart cycles in the chosen region of examination (ROE) was measured automatically with the use of software package PixelFlux (Chameleon Software, Leipzig, Germany). The Color Doppler frequency was set on 3.4 MHz and never changed. The ROE was chosen in the midsegment of renal cortex without focal changes, in the area between the outer border of medullary pyramids and the kidney surface as described by Scholbach et al. [6]. The ROE contained vessel running in straight toward the transducer and was divided into two equal segments: proximal and distal. The whole intensity of ROE was calculated as total perfusion intensity (TPI). The intensities of proximal and distal cortices were measured separately and recoded as a proximal perfusion intensity (PPI) and distal perfusion intensity (DPI), and they were then considered for statistics.

For measurement of intima-media thickness (IMT) of the left common carotid artery, the 11 L transducer (10–13 MHz) was used. Left common carotid artery was chosen because of direct aortic connection. The mean of 3 measurements of IMT in the distance of at least 10 mm before carotid sinus was calculated.

### 2.5. Cardiac Sonography

For echocardiography, the Vivid S6 (GE) with M4S-RS transducer (1.5–3.6 MHz) was used. In all patients, left ventricular mass index (LVMI) and ejection fraction (EF) were assessed and considered for statistics.

### 2.6. Statistical Analysis

The examined variables were analyzed with Spearman's correlation test as determined by missing the condition of normal distribution. The Wilcoxon test was used to analyze the difference between perfusion values. Linear stepwise regression analysis model was used to determine independent factors influencing distal perfusion intensity.

## 3. Results

**Figure 1:**
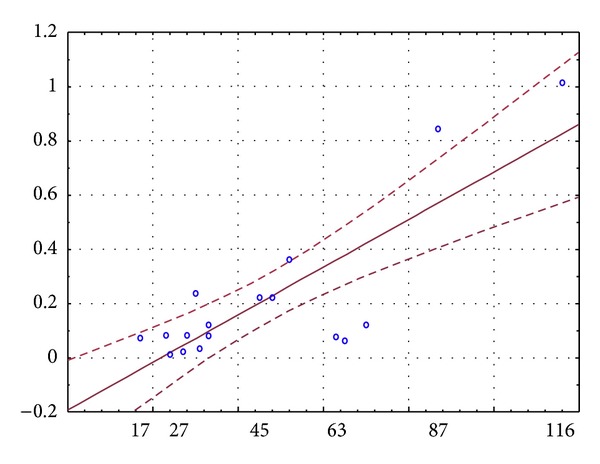
Linear regression of DPI (*y*-axis) and CKD-EPI(Cys) with 95% confidence interval.

 Results of blood sample tests are shown in Table 2. Due to CKD creatinine, cystatin C and urinary acid were elevated, and renal function in CKD-EPI equations was diminished. Results of ultrasound examinations and ABPM are presented in Table 3. In the study group, BP was well controlled, and mean 24-hour BP values were in normal range. Unsurprisingly, the mean DPI value was significantly lower than that of PPI (*P* = 0.004). Correlations of renal tissue perfusion with markers of renal function and signs of CVOD are presented in Table 4. In these results, we found a strong dependence of DPI only from biochemical markers of heart failure. In multiple stepwise regression analysis model including age, Cys, CKD-EPI(Cys), CKD-EPI(Crea-Cys), NT-proBNP, troponin I, LVMI, and IMT, only CKD-EPI(Cys) independently influenced DPI (*R* ^2^  0.67; *P* < 0.001) (Figure 1).

## 4. Discussion

In the presented study, we have shown the possibility of ultrasound quantitative evaluation of renal cortex perfusion of native kidneys. In previous publications, authors of that method of perfusion evaluation have proved its usefulness in children, in adolescents, and in patients after kidney transplant [6, 17]. Both transplanted and children kidneys are situated closer to the surface of the skin. The depth of imaging is a substantial limitation in ultrasonography, and a good visibility of the structures that lay deeper in the body needs lower frequency of ultrasounds, which can significantly affect the results of examination. Probably due to this fact, Scholbach et al. have carried out their Doppler measurements with frequency of 7 MHz, while in our study it was necessary to set the frequency twice lower, that is, 3.4 MHz [6, 17]. 

 In the presented results, total renal cortex perfusion was dependent only on age and cystatin C. This result confirms the correctness of the measurement method and corresponds to the reduction of the perfusion with aging and the decline in renal function. Also significantly lower perfusion of distal parts of renal cortex is not surprising. A similar result was obtained by Scholbach et al. who showed significantly lower values of renal cortex perfusion in its distal area [17]. This fact is a result of anatomical route of the vessels, whose diameter decreases in the direction of distal part of the cortex diminishing supply of blood. In addition, low flow values may be beyond the reach of detection by the Doppler sonography. 

 In a study of 38 transplanted kidneys, proximal to distal perfusion index was 2.99 after one year from transplantation and was progressively increasing during the next 3–5 years, and then it was decreasing, which was firstly corresponding to reduction of distal perfusion and then proximal perfusion in the course of progressive nephropathy [17]. In the earlier study of native kidneys in healthy children, PPI/DPI index was 3.64 [6]. But these results cannot be compared due to a completely different material. Limitation of distal perfusion corresponds to the function of the kidneys, reduced in our study by half. Probably due to this fact, PPI/DPI index calculated in our study was higher and amounted to 6.95. Chronic pathological processes leading to the development of CKD cause a decrease in renal perfusion, initially most expressed in distal areas of the renal cortex [7]. In some diseases, such as diabetes or metabolic syndrome, an increase of perfusion and kidneys hyperfiltration and an increase of functional tissue hypoxia are firstly observed, causing the development of inflammation with secondary fibrosis consequently limiting perfusion [1]. Although discussed perfusion disorders can be seen even before the possibility of a biochemical diagnosis of changes and can be a valuable diagnostic clue, these patients were intentionally not included in the study (exclusion criteria). In our work, perfusion of proximal region of the renal cortex did not significantly depend on renal function parameters, RRI, and markers of CVOD. Perhaps these relations will appear in a more severe renal impairment or advanced cardiovascular changes. In the study group we have found moderate reduction of renal function and discreetly elevated values of IMT and LVMI. Despite weakly expressed signs of organ damage, we have found that the quantity of distal region perfusion of the renal cortex significantly depends both on renal function measured with cystatin C and on IMT, NT-proBNP, and troponin I. We have also observed a trend of dependence of DPI on age and LVMI. It is likely that in a study on a larger group of patients DPI will prove to be a good marker of CVOD. The fact that, in almost half of the patients in our study, cardiovascular diseases were not the main cause of kidney damage may explain a higher correlation between perfusion and parameters of renal damage than CVOD markers. In the opposite situation, we can expect a significant independent effect of CVOD on the value of cortical perfusion. In previous studies, the superiority of renal function assessment by cystatin C as compared to creatinine has been demonstrated [18, 19]. This could explain the lack of correlation of renal perfusion with creatinine and its strong relation to cystatin C.

 From our study, several noteworthy conclusions can be drawn. First of all, our study has shown that quantity of renal cortex perfusion significantly correlates with the markers of cardiovascular organ damage as well as renal function. Second, a significant correlation between RRI measured in segmental arteries of the kidney and cortical perfusion has not been stated. On one hand, it seems that disorders of distal cortical perfusion reflect the level of local and systemic vascular organ damage, predisposing to recognize DPI as a sensitive marker of these pathologies. On the other hand, a lack of link between DPI and RRI is unclear and calls this thesis into question. Noncompliance can be explained by a small size of the study group and a mild severity of organ alterations influencing RRI (in this case, RRI is less sensitive), different test method (Pulsed Doppler for RRI and Color Doppler for PDI), but above all referencing of total blood flow to supplied area, and not only a measurement in clearly visible vessels, as it is in case of RRI. In other available Doppler studies, relations between renal perfusion and RRI have not been shown as well [11, 20]. Based on the obtained results, it seems that perfusion disorders of distal areas of the renal cortex are more sensitive than RRI, markers of local and systemic cardiovascular organ damage. If this assumption is confirmed in subsequent studies, DTP could be a very useful tool in the diagnostics of cardiorenal syndrome.

Despite the promising results, our study has several limitations. Too small a group and dominance of men do not allow for a generalization of the results. The drawback is the differentiation of the etiology of chronic kidney disease. In addition, patients were treated with different antihypertensive and nephroprotective drugs that could modify the value of renal perfusion. The lack of analysis of medications and their effects limits the study value. Therefore, a verifying study on a larger group of patients and without our limitations is needed.

## 5. Conclusions

The Color Doppler measurement is a reliable method of quantitative renal cortex perfusion estimation. Cardiovascular and kidney damage significantly influences renal cortical perfusion. Ultrasound measurement of renal perfusion could be a sensitive method for early investigation of cardiovascular and kidney injuries.

## Figures and Tables

**Table 1:** Diagnosis responsible for CKD in examined patients.

Diagnosis	No.
Glomerulonephritis	7
Hypertension	9
Heart failure	1

**Table 2:** Results of blood sample tests.

Data	Mean value (*n* = 17)	Standard deviation (range)
HGB (g/dL)	13.34	±1.72
Cystatin C (mg/L)	1.82	±0.64
CRP (mg/dL)	m. 0.24	(0.02–12.8)
UA (mg/dL)	7.91	±1.13
Creatinine (mg/dL)	2.04	±0.86
CKD-EPI(Crea) (mL/min/1.73 m^2^)	48.71	±30.76
CKD-EPI(Cys) (mL/min/1.73 m^2^)	46.59	±26.46
CKD-EPI(Crea-Cys) (mL/min/1.73 m^2^)	46.53	±28.01
Troponin I (ng/mL)	m. 0.015	(0.002–0.390)
NT-proBNP (pg/mL)	m. 82.80	(10.60–11799.00)

m.: median.

**Table 3:** Results of ultrasound examination and ABPM.

Data	Value (*n* = 17)	Standard deviation (range)
TPI (cm/s)	0.439	±0.365
DPI (cm/s)	m. 0.084	(0.012–1.020)
PPI (cm/s)	0.647	±0.573
RRI	0.676	±0.103
IMT (mm)	0.774	±0.255
EF (%)	59.54	±9.98
LVMI (g/m^2^)	133.75	±44.98
SBP (mmHg)	130.6	±15.3
DBP (mmHg)	78.0	±11.9
MAP (mmHg)	95.6	±11.7
PP (mmHg)	52.6	±12.57

m.: median.

**Table 4:** Correlations of total, proximal, and distal intensities of renal cortex.

Data	Intensity		
	Total	Proximal	Distal
Age	−0.581**	−0.248	−0.414^#^
HGB	−0.086	−0.242	−0.031
Creatinine	−0.396	−0.129	−0.274
Cystatin C	−0.583**	−0.289	−0.540**
CKD-EPI(Crea)	0.405	0.038	0.478^#^
CKD-EPI(Cys)	0.530**	0.205	0.586**
CKD-EPI(Crea-Cys)	0.440^#^	0.115	0.505**
Uric acid	−0.020	−0.117	−0.016
CRP	−0.037	0.027	−0.266
NT-proBNP	−0.436^#^	−0.110	−0.607**
Troponin I	−0.201	0.049	−0.535**
EF	0.066	0.037	−0.063
LVMI	−0.103	0.083	−0.428^#^
RI	−0.476^#^	−0.332	−0.291
IMT	−0.618**	−0.283	−0.558**
SBP	0.177	−0.075	0.089
DBP	0.362	0.041	0.170
MAP	0.428^#^	0.118	0.196
PP	−0.070	−0.199	−0.208

**Significance *P* < 0.05; ^#^trend *P* < 0.1 for Spearman's coefficient.
